# Cell Proliferation and Migration Are Modulated by Cdk-1-Phosphorylated Endothelial-Monocyte Activating Polypeptide II

**DOI:** 10.1371/journal.pone.0033101

**Published:** 2012-03-08

**Authors:** Margaret A. Schwarz, Janet Thornton, Haiming Xu, Niranjan Awasthi, Roderich E. Schwarz

**Affiliations:** Departments of Pediatrics and Surgery, University of Texas Southwestern Medical Center at Dallas, Dallas, Texas, United States of America; National Cancer Institute, United States of America

## Abstract

**Background:**

Endothelial-Monocyte Activating Polypeptide (EMAP II) is a secreted protein with well-established anti-angiogenic activities. Intracellular EMAP II expression is increased during fetal development at epithelial/mesenchymal boundaries and in pathophysiologic fibroproliferative cells of bronchopulmonary dysplasia, emphysema, and scar fibroblast tissue following myocardial ischemia. Precise function and regulation of intracellular EMAP II, however, has not been explored to date.

**Methodology/Principal Findings:**

Here we show that high intracellular EMAP II suppresses cellular proliferation by slowing progression through the G2M cell cycle transition in epithelium and fibroblast. Furthermore, EMAP II binds to and is phosphorylated by Cdk1, and exhibits nuclear/cytoplasmic partitioning, with only nuclear EMAP II being phosphorylated. We observed that extracellular secreted EMAP II induces endothelial cell apoptosis, where as excess intracellular EMAP II facilitates epithelial and fibroblast cells migration.

**Conclusions/Significance:**

Our findings suggest that EMAP II has specific intracellular effects, and that this intracellular function appears to antagonize its extracellular anti-angiogenic effects during fetal development and pulmonary disease progression.

## Introduction

Endothelial Monocyte Activating Polypeptide II (EMAP II, also known as AIMP-1, Scye-1, and p43), a single chain polypeptide protein originally isolated from a murine fibrosarcoma, is ubiquitously expressed as a 34-kDa intracellular protein [1]. On the cell surface, it undergoes proteolytic cleavage [2], [3] to generate a ≈22-kDa C-terminal peptide [4], [5], [6] that functions as a potent anti-angiogenic protein [1], [7]. As an extracellular molecule, C-terminal EMAP II (ct-EMAP II) is known to activate endothelial cells, neutrophils and mononuclear phagocytes [5], [6]; consequently, ct-EMAP II has shown the capacity to prime tumor vasculature for a locally destructive process, or to be anti-angiogenic in its own capacity [1], [8], [9], [10]. Mechanistically ct-EMAP II suppresses primary and metastatic tumor growth through inhibition of endothelial cell adhesion to fibronectin [11], disrupts alveolar epithelial type II to type I cell transdifferentiation [12], regulates pulmonary cell-cell cohesion, aggregation, and lung assembly [13], blockade of fibronectin matrix assembly via α5β1 integrin [9], [11], and interference with vascular endothelial growth factor (VEGF) induced pro-angiogenic signaling [14].

Based on a high degree of homology between EMAP II and the aminoacyl-tRNA synthetase (ARS) p43, EMAP II is considered a member of the larger ARS family [15]. ARSs are proteins that catalyze ligation of their cognate amino acids to specific tRNAs. Although the basic core domain is well conserved among the ARSs, N- or C- terminal residues on p43 suggest that it is likely to mediate additional functions beyond amino acid loading of tRNAs. P43 is one of three auxiliary proteins (p38, p18, and p43), with p38 having a pivotal role in the assembly of the subunits of the eukaryotic tRNA synthetase complex [16]. Interestingly, the extracellular function of p43 is similar to EMAP II in that it possesses extracellular anti-angiogenic activities [17], while its intracellular role beyond that of an RNA binding protein remains less well understood [18].

Here, we examine the intracellular role for EMAP II. Previously, we and others have found high levels of EMAP II at epithelial/mesenchymal cell interfaces of proliferating and differentiating cells in embryonic lungs [19], [20], [21], within dysplastic fibroproliferative regions of bronchopulmonary dysplasia (BPD) [22] and in emphysematous lungs [23], suggesting that EMAP II might regulate cell proliferation in organ development and disease. We demonstrate that overexpression of intracellular EMAP II delays cellular proliferation and progression through the G2M phase of cell cycle. Furthermore, EMAP II expression increases during the cell cycle at the S and G2M phases, leading to its phosphorylation. Intracellular EMAP II exhibits distinct nuclear/cytoplasmic partitioning and associates with Cdk1 in the N-terminal region. In conjunction with the delay in cell cycle and reduced proliferation, there is a marked increase in cellular motility. These studies indicate that EMAP II possesses an intracellular role that may influence cellular proliferation and migration during fetal development and in pulmonary disease progression.

## Results

### Overexpression of EMAP II reduces cell proliferation and delays G2M exit in cell cycle

**Figure 1:**
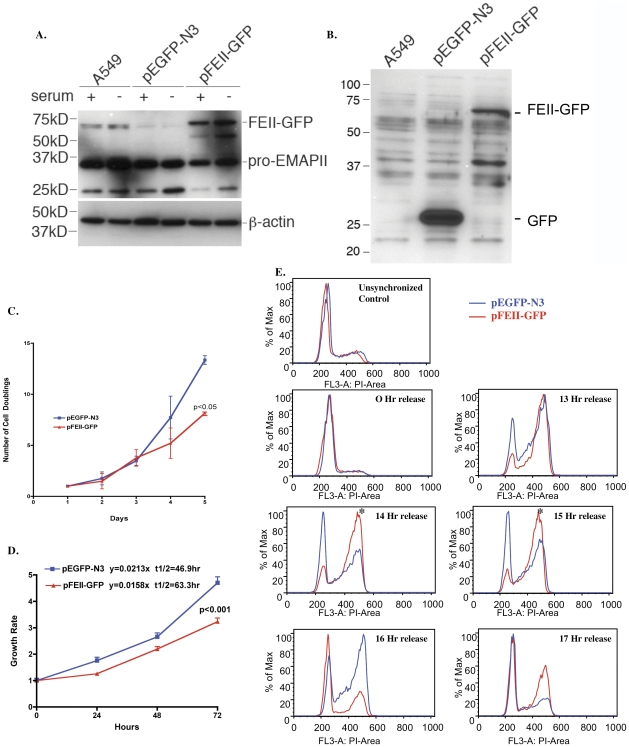
EMAP II overexpression delays cell proliferation. Cell doubling, proliferation and progression through cell cycle were examined in clonal A549 cell populations overexpressing either EMAP II (pFEII-GFP) or GFP (pEGFP-N3). Western analysis of cell lysates using either EMAP II (A) or GFP (B) antibody confirmed expression. EMAP II overexpressing cells were found to have a marked delay in cell doubling (C, p<0.05), growth rate (D, p<0.001), and a delay in exiting the G2M phase of cell cycle as noted at 14 and 15 hours post synchronized cell release (*, E) as compared to the empty vector control (pEGFP-N3) (these experiments were performed a minimum of 4 times).

To determine the role of intracellular EMAP II in proliferating cell populations, stable clonal A549 cell populations that overexpressed EMAP II protein (pFEII-GFP) or an empty vector (pEGFP-N3) were established using antibiotic pressure selection (WB of whole cell lysate, Figure 1A and 1B). Overexpression of EMAP II delayed cell doubling times (Figure 1C, p<0.05, ANOVA, representative experiment, n = 6, performed on 3 different occasions) and growth rate (Figure 1D, WST-1, p<0.001, ANOVA, representative experiment, n = 4 (in triplicate), performed on 4 different occasions) as compared to an empty vector control. Delays in doubling time and growth rate were not due to induction of apoptosis as there were no differences between apoptosis-related proteins in form of PARP-1 cleavage and phospho-JNK cellular protein as measured by WB or detection of early apoptosis and annexin/PI via FACs analysis (data not shown) between the two clonal cell populations. As EMAP II's ability to reduce proliferation and doubling times was not secondary to apoptosis, the impact of EMAP II overexpression on cell cycle was explored. Progression from the G0 phase of cell cycle was examined in chemically synchronized pEGFP-N3 and pFEII-GFP cells. At 0 hour release, greater than 80% of both clonal cell populations were in the G0 phase of cell cycle. Cells progressed through cell cycle similarly in the early phases G1 and S (Figure 1E, 13 hour). In contrast, cells overexpressing EMAP II (pFEII-GFP, noted in red, Figure 1E) had a marked delay in G2M progression that persisted for 2 hours noted between 14 and 15 hours (representative experiment, n = 6 performed on different occasions).

### EMAP II over-expression increases cell migration

**Figure 2:**
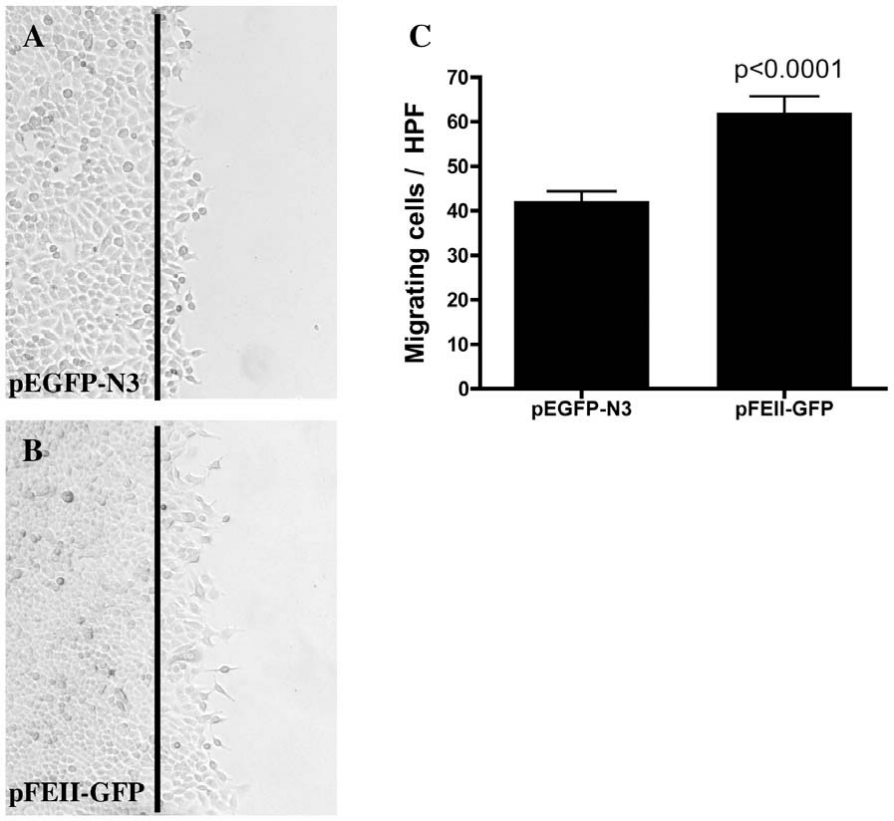
Overexpression of EMAP II increases cell migration. Confluent cells were wounded using a rubber policeman, wounding site marked, and examined for migration 15 hours post wounding. The black line represents the wounded margin. Cells overexpressing pFEII-GFP (B,C) had a marked increase in migration as compared empty vector control (pEGFP-N3, A,C) (p<0.0001) (n = 9 in triplicate performed on 3 different occasions).

To assess potential effect of high intracellular EMAP II on cell migration, in vitro scratch migration assay was performed using A549 cells over-expressing EMAP II. As illustrated in Figure 2A & 2B, over-expression of EMAP II induced cell migration. Quantitation of migratory cells by light microscope demonstrated a marked increase in cell migration in EMAP II over-expressing cells (50 percent increase) (Figure 2C, p<0.0001, unpaired Student t-test, n = 9 in triplicate performed on 3 different occasions) as compared to control. To confirm that there no cellular proliferation was occurring in the migrating cell population, DAPI immunofluorescent staining was utilized. No proliferation was noted in either cell population at the wound margin (data not shown).

### Nuclear/cytoplasmic partitioning of EMAP II in NIH 3T3 cells

**Figure 3:**
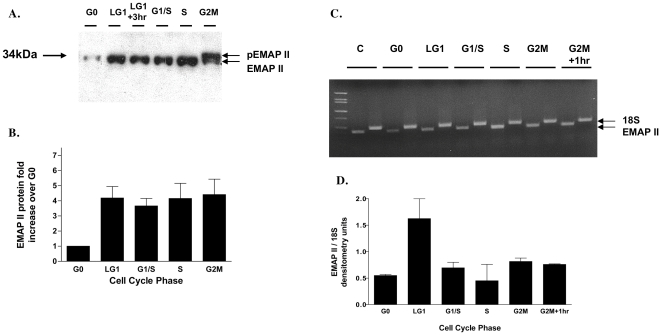
EMAP II is phosphorylated at G2M phase of cell cycle. EMAP II translation and transcription were examined in chemically synchronized cells throughout the phases of cell cycle. Western blot analysis indicated that upon exiting G0, EMAP II protein was increased throughout cell cycle and was phosphorylated at G2M (A,B). EMAP II transcription was minimally impacted during the different phases of cell cycle (C,D) (these experiments were performed a minimum of 4 times).

As overexpression of EMAP II was noted to delay cellular proliferation and cell cycle progression, we explored EMAP II expression in the different phases of cell cycle. In chemically synchronized NIH 3T3 cells, cell-cycle phases were examined for EMAP II transcriptional and translational expression throughout the different phases of the cell cycle (Figure 3A–D). EMAP II protein expression increased 4-fold from G0 to the late G1 phase of cell cycle and remained elevated through G2M (Figure 3A and 3B, p = 0.02, ANOVA, n = 4 on 4 different occasions). Importantly, at G2M cycle point EMAP II was phosphorylated (pEMAP II) (Figure 3A, arrow pEMAP II). EMAP II RNA levels, standardized to 18S, were transiently elevated 3 fold at late G1 prior to returning at the G1/S to levels (Figure 3C and 3D) (n = 6, performed on 6 different occasions).

**Figure 4:**
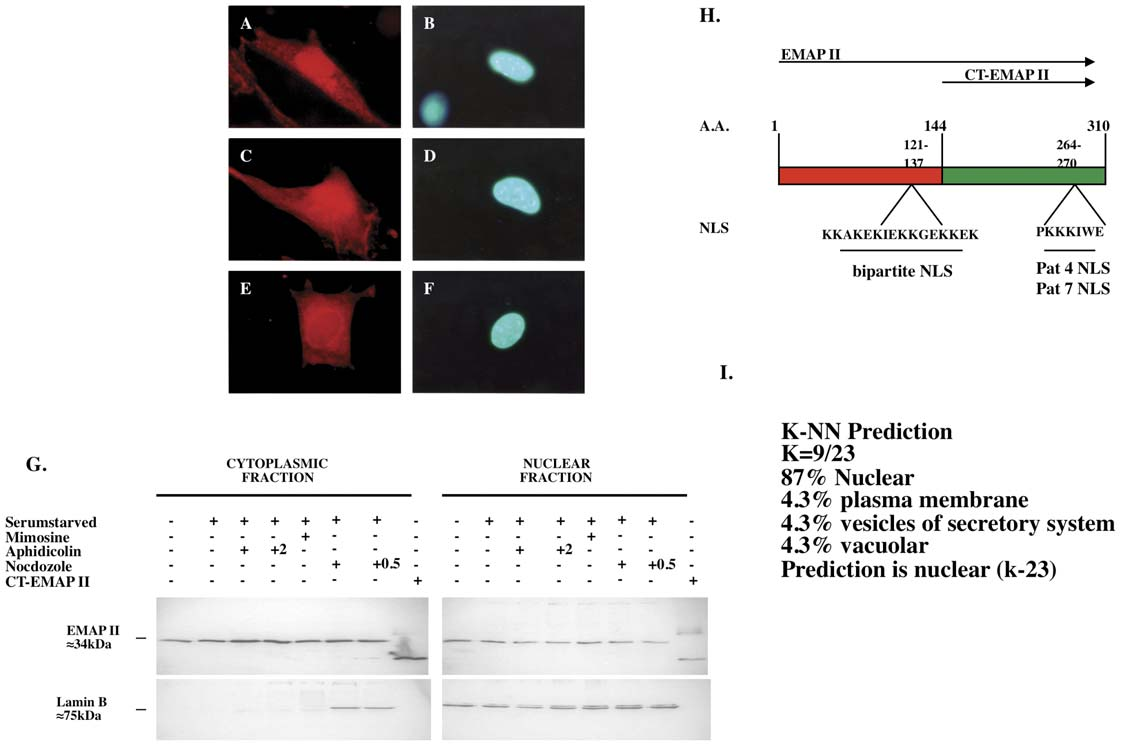
EMAP II undergoes nuclear/cytoplasmic partitioning during cell cycle. EMAP II distribution was assessed in chemically synchronized cell populations. Immunofluoresence indicated EMAP II expression is predominately nuclear in G0 (A,B) and S (C,D) phase, whereas in G2M (E/F) nuclear EMAP II is reduced. Western blot analysis (G) of isolated nuclear and cytoplasmic fractions indicates EMAP II expression is maintained in both compartments through out cell cycle (these experiments were performed 4 times). PSORT II analysis determined there were three classical types of nuclear localizing sequences (NLS): pat4, pat7, and a bipartite NLS (H). Proteins K and R composition predicts that 87% of EMAP II is subcompartmentalization to the nucleus EMAP II's (I). Magnification in A–F: 1000×.

To determine EMAP II cellular location during cell cycle phases, chemical synchronization and immunofluorescence using an N-terminal EMAP II antibody during G0, S, and G2M of cell cycle was performed. EMAP II was identified in both cytoplasm and nuclear locations, where its immunofluorescent expression was predominately nuclear from G0 through S phase of cell cycle (Figure 4A and 4C, respectively) followed by a reduction in nuclear expression at G2M (Figure 4E) (n = duplicates performed on 4 different occasions). Cytoplasmic and nuclear isolates from synchronized NIH3T3 cells confirmed presence of EMAP II in both fractions, but did not indicate significant nuclear–cytoplasmic shifts in EMAP II during cell cycle (Figure 4G). Interestingly, EMAP II's nuclear location was found to be consistent with a PSORT II analysis (http://psort.nibb.ac.jp/psort) indicating that EMAP II contains three classical types of nuclear localizing sequences (NLS) (Figure 4H). The first, pat4, is composed of 4 residue patterns of basic amino acids (K or R) and is located at the aa 264–270 region of EMAP II. A second NLS, pat7, was noted by a pattern starting with P that is subsequently followed within 3 residues by a basic segment containing 3 K/R residues out of 4. This region is also located in the aa 264–270 region. Lastly, a bipartite NLS containing 2 basic residues, 10-residue spacer, and a second basic region consisting of at least 3 basic residues out of 5 were identified in region aa 121–137 of EMAP II. Utilizing protein K and R compositions, one can predict EMAP II's subcompartmentalization. Computation of EMAP II's K and R composition predicts that 87% of EMAP II is localized to the nucleus (Figure 4I).

### EMAP II is phosphorylated at amino acid number 84, threonine, by cdk1, and is compartmentalized in the nucleus

**Figure 5:**
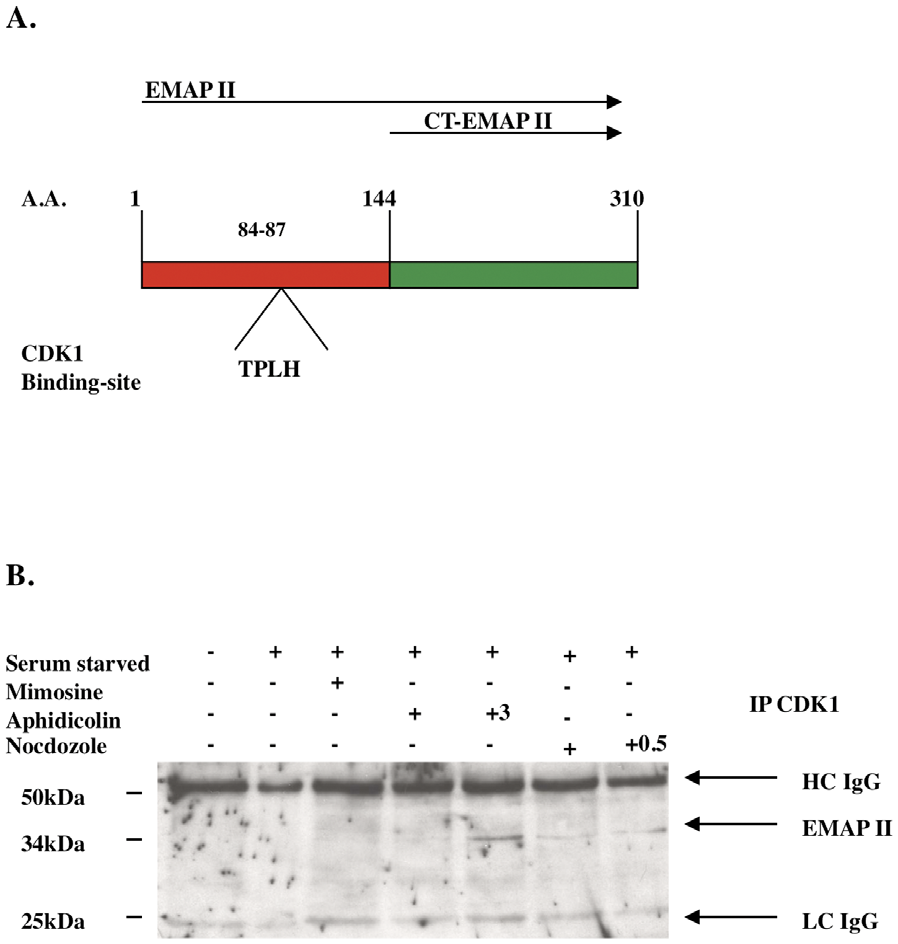
EMAP II contains a Cdk1 binding site and immuno-precipitates with Cdk1 during S and G2M phases of cell cycle. A Cdk1 binding domain, TPLH, was identified in EMAP II's N-terminal region at aa 84–87 (A). Immunoprecipitation using Cdk1 determined that EMAP II binds to Cdk1 during S and G2M phase of cell cycle (B) (these experiments were performed 4 times).

**Figure 6:**
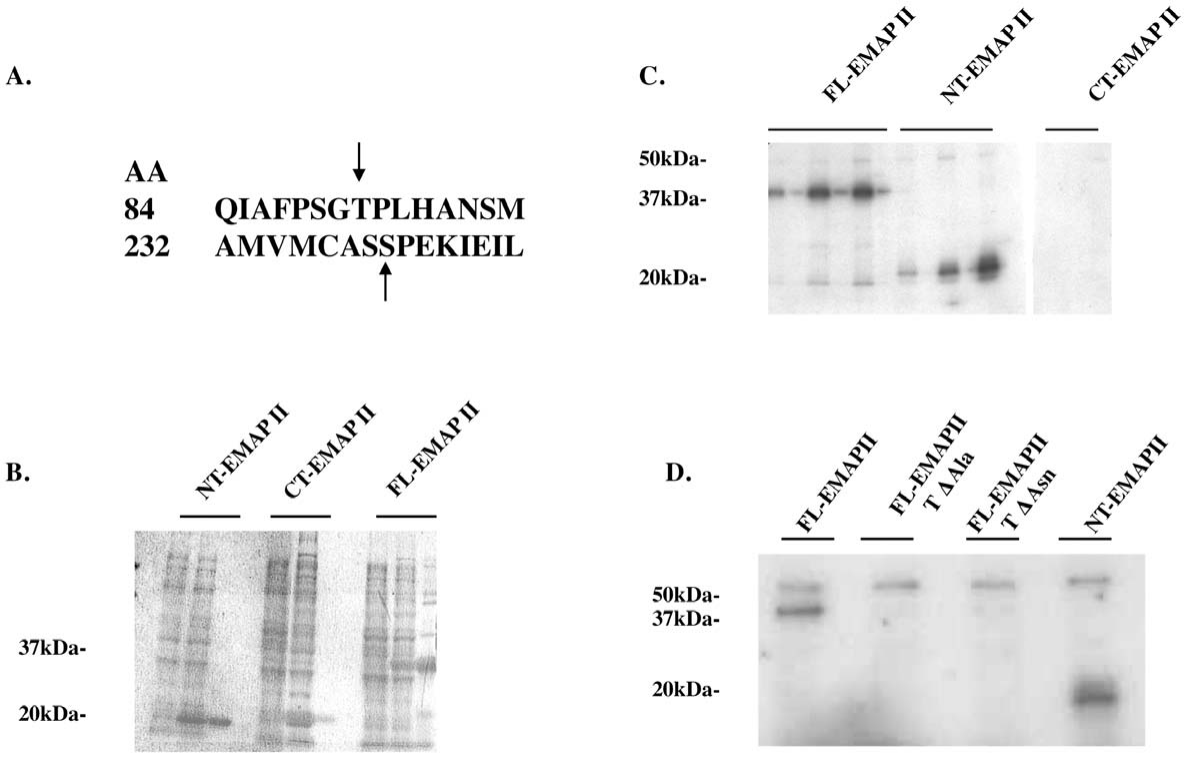
Cdk1 phosphorylates EMAP II at amino acid 84, threonine. Two potential Cdk1 phosphorylation sites were identified, aa 84 threonine, and serine at aa 232 (A). Using recombinant EMAP II protein isolates (B) in a Cdk1 assay, only the N-terminal and full-length EMAP II were phosphorylated by Cdk1 (C). Point mutation within the threonine (aa 84) to change the amino acid to either asparagine or alanine eliminated Cdk1 phosphorylation, thus confirming that the 84 aa threonine was the site of Cdk1 phosphorylation of EMAP II (D) (these experiments were performed 3 times).

**Figure 7:**
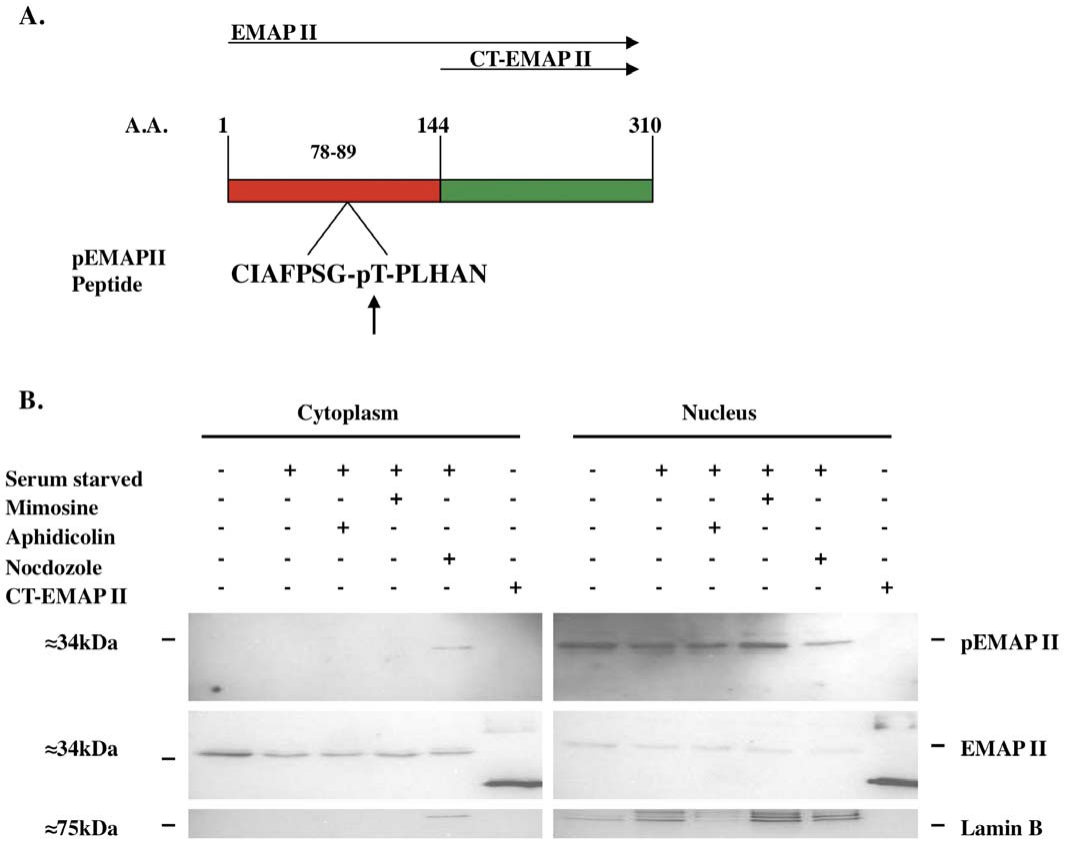
Phosphorylated EMAP II is predominately localized in the nuclear fraction. A phosphorylated pThr84-EMAP II antibody was obtained (A) and phospo-EMAP II was examined in nuclear and cytoplasmic fractions during cell cycle. Phospho-EMAP II was found in the nuclear fraction G0-S phase of cell cycle with only the phosphorylated form found in the cytoplasm during G2M. Unphosphorylated EMAP II (capable of recognizing unphosphorylated and phosphorylated EMAP II) was confined to the cytoplasm as only a single band corresponding with the phosphorylated form was found in the nuclear isolates (B). Lamin B nuclear envelope confirmed nuclear isolate specificity (these experiments were performed a minimum of 4 times).

As Cdk1 has a prominent role in controlling progression through the G2M cell cycle phase, and since a Cdk1 binding domain was identified in EMAP II's N-terminal region (aa 84–87, TPLH, Figure 5A), binding of Cdk1 to EMAP II was examined. Immunoprecipitation with Cdk1 using whole cell lysates indicate that EMAP II binds to Cdk1 during the G2M transition (determined by FACs of cells simultaneously plated) (Figure 5B; n = 4 performed on 4 different occasions). Co-immunofluorescence of EMAP II and Cdk1 suggests that during G0 phase (Figure S1A, S1B, S1C) EMAP II and Cdk1 are expressed in the cytoplasm and nucleus with predominate nuclear expression by both in S phase (Figure S1D, S1E, S1F). In contrast, during G2M, EMAP II is predominately cytoplasmic (Figure S1G, S1I) with Cdk1 being both nuclear and cytoplasmic (Figure S1H, S1I). Phosphorylation of EMAP II at G2M suggests that it may be interacting with one of the prominent kinases that has been identified as facilitating the transition of cells into mitosis, Cdk1, a serine/threonine kinase. Motif scanning (http://scansite.mit.edu) of the EMAP II sequence identified two potential sites for Cdk1 phosphorylation, threonine at amino acid number 84, and serine located at the 232 amino acid site (Figure 6A). Utilizing a recombinant bacterial system three different EMAP II isoforms [FL (full length) EMAP II, CT (C-terminal) EMAP II and NT (N-terminal) EMAP II] were utilized to evaluate potential phosphorylation candidates. Electrophoresis analysis performed on isolates gathered during the purification process indicated that the recombinant his-tagged purified recombinant EMAP II protein isoforms were soluble (Figure 6B). Cdk1 assay performed on recombinant EMAP II isoforms suggest that FL-EMAP II and NT-EMAP II, but not the CT-EMAP II, were phosphorylated by Cdk1 indicating that the site of phosphorylation was located in the N-terminal region of EMAP II, threonine at 84. Specificity of Cdk1 phosphorylation was confirmed when FL-EMAP II and NT-EMAP II were phosphorylated by Cdk1, but point mutations introduced at the threonine 84 amino acid site to change the threonine to alanine (FL-EMAP II TΔAla) or asparagine (FL-EMAP II TΔAsn) were not phosphorylated by Cdk1 (Figure 6D) (n = 9 performed on 3 different occasions). These data suggest that EMAP II binds to Cdk1 and is phosphorylated at the threonine aa84 by Cdk1 during G2M of cell cycle. Using an pThr84-EMAP II antibody, we examined the distribution of phospo-EMAP II during cell cycle. In contrast to our previous analysis of cell lysate, we determined that phospho-EMAP II is predominately nuclear while unphosphorylated EMAP II is cytoplasmic (Figure 7).

## Discussion

Intracellular EMAP II expression is increased during fetal development at epithelial/mesenchymal boundaries [19], [21] and in pathophysiologic fibroproliferative processes such as the distal alveoli of BPD [22], emphysema [23], and the scar tissue of myocardial ischemia [24]; the functional role of intracellular EMAP II, however, is poorly understood. EMAP II's pathophysiologic distribution raised the possibility that EMAP II may have a role in cellular proliferation. Overexpression of intracellular EMAP II delayed proliferation of epithelial cells, doubling, and transition through the G2M phase of the cell cycle without inducing cellular apoptosis, while increasing cell migration. Based on a recent study, some anti-angiogenic proteins cleaved from larger precursor proteins can demonstrate separate and independent intracellular functions that antagonize the cleaved anti-angiogenic form [25]. For example, the anti-angiogenic protein endostatin is cleaved from the larger secreted extracellular matrix protein Collagen XVIII. Whereas endostatin inhibits endothelial cell proliferation, migration and induces apoptosis, collagen XVIII positively regulates the extracellular matrix-dependent motility and morphogenesis of endothelial cells. This dichotomous coupling of a vascular agonist and antagonist, with its dependency on the proteolytic cleavage of one protein, suggests that there is an autoregulatory feedback loop between the two isoforms [25].

In addition, examination of EMAP II expression and distribution during the cell cycle progression revealed an elevation in EMAP II protein during G1-G2M stages of cell cycle with EMAP II phosphorylation, nuclear and cytoplasmic distribution, binding and phosphorylation to Cdk1, and phosphorylated EMAP II being confined to the nuclear isolates. These findings indicate that EMAP II has an intracellular role affecting cellular proliferation and migration, i.e. acting as an agonist to which its extracellular role carries an anti-angiogenic antagonistic function.

Cell cycle progression is controlled by a series of carefully regulated signals that involve protein phosphorylation. For example, the regulatory activity of cdc2/Cdk1 (cyclin dependent kinase 1), a primary determinant of mitosis, is dependent on its phosphorylation state. Inhibitory phosphorylation of Cdk1 restrains it to an inactive state. Once dephosphorylated, Cdk1 is activated to facilitate the mitosis transition through reorganization of cellular structures, chromosome condensation, disassembly of the nuclear envelope, and assembly of the mitotic spindle [26]; [27]. Inhibition of Cdk1 dephosphorylation maintains its inactive state and blocks cell cycle progression at the G2/M transition [28], [29]. We determined that EMAP II complexes to Cdk1 during the G2M phase and is phosphorylated by Cdk1. Furthermore, phosphorylated EMAP II was confined to the nucleus and its overexpression delayed G2M progression. These findings raise several possible mechanisms. In cell cycle progression EMAP II may either simply carry the phosphate that allows retention of active Cdk1 longer, or be activated to partake in cell cycle regulation itself. Dissecting out the mechanism of EMAP II-related delay of G2M progression is the primary focus of our ongoing studies.

Following tissue injury, remodeling occurs that is mediated through inflammatory, trans-differentiation, and apoptotic mechanisms. In contrast to CT-EMAP II's well-documented extracellular pro-inflammatory roles [30], [31], [32], [33], the functional role of intracellular EMAP II in pathophysiologic states has remained poorly understood. Intracellular expression of EMAP II is increased in fibroproliferative regions of disease states such as the BPD dysplastic alveoli [22] or the wound region of an infarcted myocardium [24] suggesting a potential physiologic role in wound repair. While apoptosis is a hallmark of many disease process and tissue repair, intracellular overexpression of EMAP II was not found to increase apoptosis via PARP-1 cleavage or JNK pathway. One previous study suggests that intracellular EMAP II is not pro-inflammatory, but rather suppressive of fibroblast proliferation in wound healing studies in mice with depletion of EMAP II/p43 expression using gene disruption [31]. Their findings are consistent with our findings that EMAP II has a specific regulatory role in cell proliferation. However, wound healing requires reciprocal events involving cellular migration and proliferation. Initial stages of wound healing involve the recruitment of immune cells and cellular migration, while macrophages contribute to later stages of reconstruction through the secretion of factors that regulate epithelial and fibroblast cellular migration and proliferation. Previous studies indicate that EMAP II expression is markedly increased in the wounded region [24] with migrating macrophages also strongly expressing EMAP II. In our studies EMAP II overexpression significantly increased cellular migration while suppressing proliferation suggesting that EMAP II may have an impact on the microenvironment and cellular components that restore tissue homeostasis and balance the tissues repair response. Whether EMAP II's ability to delay cellular proliferation while increasing cellular migration impacts trans-differentiation during tissue remodeling is an area of ongoing research within our laboratory.

In conclusion, our studies show for the first time a functional role for intracellular EMAP II where EMAP II is phosphorylated by Cdk1 in the nuclear compartment and influences cellular proliferation and migration in a reciprocal fashion. Furthermore, these findings suggest that the intracellular function of EMAP II possibly antagonizes its extracellular anti-angiogenic functions during fetal development [19], [21], pulmonary disease progression [22], [23], [34], and wound healing [24]. These findings broaden our current understanding of the spectrum of regulatory roles for EMAP II in development and wound healing processes.

## Materials and Methods

### Cells

NIH3T3 fibroblasts and carcinomic human alveolar basal epithelial cell line A549 cells, purchased from ATCC (Manassas,VA), were grown in DMEM, 10% CBS (calf bovine serum, ATCC, Manassas,VA) 1% Penicillin/Streptomycin (Sigma, St. Louis, MO) and 5% CO_2_. Cells were passaged every 3–4 days utilizing trypsinization techniques.

### Construction of human EMAP II-GFP fusion plasmid, pFEII-GFP

The open reading frame of full-length EMAP II was amplified using a high fidelity PCR kit (Sigma Aldrich, St. Louis, MO) from previously constructed plasmid pET28a FEII [34]. The PCR product was digested with Xho I plus Hind III and inserted into vector pEGFP-N3 cut with Xho I plus Hind III. Plasmid accuracy was verified with restriction digestion and DNA sequencing.

### Establishment of A549 Stable clones

A549 cells were maintained in our lab and cultured in DMEM containing 10% fetal bovine serum at 37°C with 5% CO_2_. A549 cells were transfected with plasmid pEGFP-N3 and pFEII-GFP using TransIT LT1 (Mirus Bio, Madison, WI) transfection reagent. After 24 hours, A549 cells were split at 1∶20 into 10 cm dishes. A549 stable clone cells were selected using 900 mcg/ml of G-418 for 2–3 weeks. GFP-positive cells were picked for further analysis under fluorescence microscope. Overexpression of GFP and EMAPII GFP fusion protein were confirmed using Western Blotting analysis.

### Cell growth curves, Proliferation and Migration studies

Stably transfected A549 cell lines, A549 vector control (pEGFP-N3) and overexpressing EMAP II (pFEII-GFP) were plated in duplicate at 0.25×10^6^ cells/ml, and allowed to grow in G418 800 µg/ml (Invitrogen, Carlsbad, CA). Cells were collected and counted on days 1–5. For proliferation assays, pEGFP-N3 and pFEII-GFP cells were plated in 6 wells of a 96-well plate at a concentration of 1000 cells/well in G418 800 µg/ml. Cells were allowed to grow for 5 days. Each day 1–5, 10 µl of proliferation reagent WST-1 (Roche, Indianapolis, IN) was added to each of the 6 wells. Plates were placed in a 5% CO_2_, 37°C incubator for one hour and then were read by POLARstar Omega - Fluorescence Polarization microplate reader (BMG Labtech, Cary, NC) at 450 nm to assess cell proliferation. Migration studies were performed in 6 well plates. Once cells reached confluence, a wound was created using a sterile rubber policeman. Sites of wounding were marked at time of initiation and migration was assessed 14–16 hours post wounding. Following image capturing using light microscopy, cells were fixed with paraformaldehyde and stained with the nuclear stain DAPI (4′,6-Diamidino-2-phenyindole 5 mg/ml at 1∶1,000 dilution, Sigma Aldrich, St. Louis, MO) to assess for cellular proliferation. A blinded observer performed migration determined as the number of cells that migrated past the wound edge.

### Chemical synchronization

Asynchronized cells were grown in 10% fetal bovine serum (FBS) at 50% confluence prior to starvation conditions (0.5% FBS) for 3 days to achieve a G0 state. For late G1, cells were exposed to 400 mM mimosine for 24 hours. Exposure of cells to twenty-four hour of Aphidicolin 2 µg/ml placed the cells at the G1/S junction. S phase was achieved using a G1/S junction delay (Aphidicolin) plus 4 hours release (confirmed with FACS analysis). The G2/M boundary was reached using nocodazole 100 ng/ml for 24 hours. For evaluation of cell cycle progression, stably transfected A549 pEGFP-N3 and pFEII-GFP clonal were allowed to grow to 50% confluence and switched to starving conditions (no FBS) for 48 hrs. Cell arrest was accomplished by adding 8 µg/ml aphidicolin (Sigma Aldrich, St. Louis, MO) in DMEM with FBS for 24 hrs. To see differences in how cells enter/leave G2, aphidicolin treated medium was removed and cells were collected for FACS analysis at 0,12,13,14,15,16,17 hours following release.

### FACs analysis

Following trypsinization, cells were centrifuged, washed with PBS, and fixed while vortexing with 70% ethanol. Fixed cells were washed with PBS and treated with RNAse 20 µg/ml (Sigma Aldrich, St. Louis, MO) at 37°C for 1 hour. Cells were stained with propidium iodine (PI) (20 µg/ml, Sigma Aldrich, St. Louis, MO). Some cells were preincubated with annexin V-FITC followed by PI prior to FACs analysis. Fluorescence of cells was measured at 488 nm by FACS Calibur (Becton Dickinson, Franklin Lakes, NJ) at the Flow Cytometry Core Facility at UTSW Medical Center.

### Recombinant protein

Recombinant EMAP II protein was obtained as previously described [34]. The cDNA of EMAP II was cloned from RT-PCR products of U937 cells total RNA based on primers obtained from gene bank (accession #10119) into TA vector (Invitrogen, Carlsbad, CA). Clonal confirmation was provided by sequence analysis, after which the cDNA was inserted into PET28a, 6× his-tag containing plasmid, protein purified as previously described [34]. Point mutations were made in EMAP II at amino acid position 84 where the threonine was mutated to an alanine or asparagines using QuikChange Site-Directed Mutagenesis Kit (Stratagene, Santa Clara, CA) and confirmed by sequence analysis.

### Western Blot Analysis

A549 cells were harvested and washed with ice-cold PBS twice. Cell pellets were resuspended in lysis buffer, protein concentration was measured using BCA method (Biorad, Hercules, CA), and equal amounts of protein were subjected to SDS-PAGE and transferred onto PVDF membrane. After blocking membranes were probed with the appropriate primary antibody [EMAP II [34], pEMAP II (YenZym Antibodies, San Francisco, CA), Cdk1 (Abcam, Cambridge, MA), Lamin B (Abcam, Cambridge, MA), JNK (Santa Cruz Biotechnologies, Santa Cruz, CA), poly (ADP-ribose) polymerase-1 (PARP-1) (Cell SignalingTechnology, Beverly, MA), GAPDH (Sigma Aldrich, St. Louis, CA)]. Specific binding was detected using a chemiluminescence substrate (Pierce, Rockford, IL) and XAR-5 film (Eastman Kodak, Rochester, NY). Quantitative analysis was accomplished using NIH Image and samples were normalized for background. In some cases, following lysis of cells, equal amounts of protein were added to tubes containing the antibody for immunoprecipitation (IP), incubated, followed by the addition of washed Protein A beads. Beads were washed, electrophoresed, and processed as described above. Immunoprecipitation was performed on 200 µg of total protein and each experiment contained internal controls (no protein A beads, no primary antibody, and no lysate). For some studies, nuclear and cytoplasmic fractions were isolated using NE-PER Nuclear and Cytoplasmic Extraction Kit (Pierce) as per manufacture protocol prior to subjecting samples to electrophoresis. Nuclear and cytoplasmic specificity was confirmed using the Lamin B nuclear envelope marker. pThr84-EMAP II antibody was created using a synthesized phosphorylated EMAP II peptide and purified using an affinity matrix purification (YenZym Antibodies, San Francisco, CA).

### Kinase assay

Target proteins were incubated with 15 µci γ - 32P dATP, 1 mM ATP (100 µm/reaction) and 4 units Cdk1 for 1 hour at 30° Celsius. Samples were then electrophoresed on a 12% Tris-Glycine gel, transferred to a PVDF membrane and exposed to an autoradiography overnight. Concomitant controls were performed without kinase as a substrate [35].

### Immunohistochemistry analysis

Cells plated on plastic chamber slides were synchronized as described above. Cells were then washed in PBS prior to fixation with 4% paraformaldehyde for 15 min. Following permeabilization with 0.1% Triton-X, cells were washed with PBS, blocked using CSA (Zymed, San Francisco, CA) and exposed to a C-terminal EMAP II primary antibody for 1 hour as previously described [20]. Following washing with PBS, cells were exposed to the appropriate secondary fluorescent antibody (Chemicon, Temecula, CA), incubated with membrane permeable DAPI (4′,6-Diamidino-2-phenyindole 5 mg/ml at 1∶1,000 dilution, Sigma Aldrich, St. Louis, MO), and mounted. In some cases, co-localization was performed using the C-terminal EMAP II antibody followed by exposure to the appropriate secondary antibody. This was followed by exposure to a Cdk1 antibody and then appropriate secondary antibody. The signal was viewed by fluorescent microscopy at the appropriate wavelength for the secondary antibody on a BX50 Olympus microscope, and images were obtained using an Olympus DP70 digital camera.

### Statistics

Statistical analysis was performed using Student's t-test and one way ANOVA on software Statview for Macintosh (SAS Institute, Gary, NC) and PRISM 4.0 for Macintosh (GraphPad Software, Inc., San Diego, CA). P-values of <0.05 were considered to represent statistically significant differences.

## Supporting Information

Figure S1 **EMAP II and Cdk1 co-localize throughout cell cycle.** Distribution of EMAP II and Cdk1 were examined throughout cell cycle using co-immunofluorescence. During G0 (A–C) EMAP II (A) and Cdk1 (B) were expressed in the cytoplasm and nucleus with a transition to a predominate nuclear expression in S phase (D–F). In contrast, during G2M EMAP II is predominately cytoplasmic (G,I) with Cdk1 being both nuclear and cytoplasmic (H,I). Magnification: 600× (these experiments were performed a minimum of 3 times).
(TIF)
