# Supplementary figures and images for: Cell Proliferation and Migration Are Modulated by Cdk-1-Phosphorylated Endothelial-Monocyte Activating Polypeptide II
Source: PLoS One. 2012 Mar 8;7(3):e33101. doi: 10.1371/journal.pone.0033101 (PMC3297626; doi:10.1371/journal.pone.0033101)

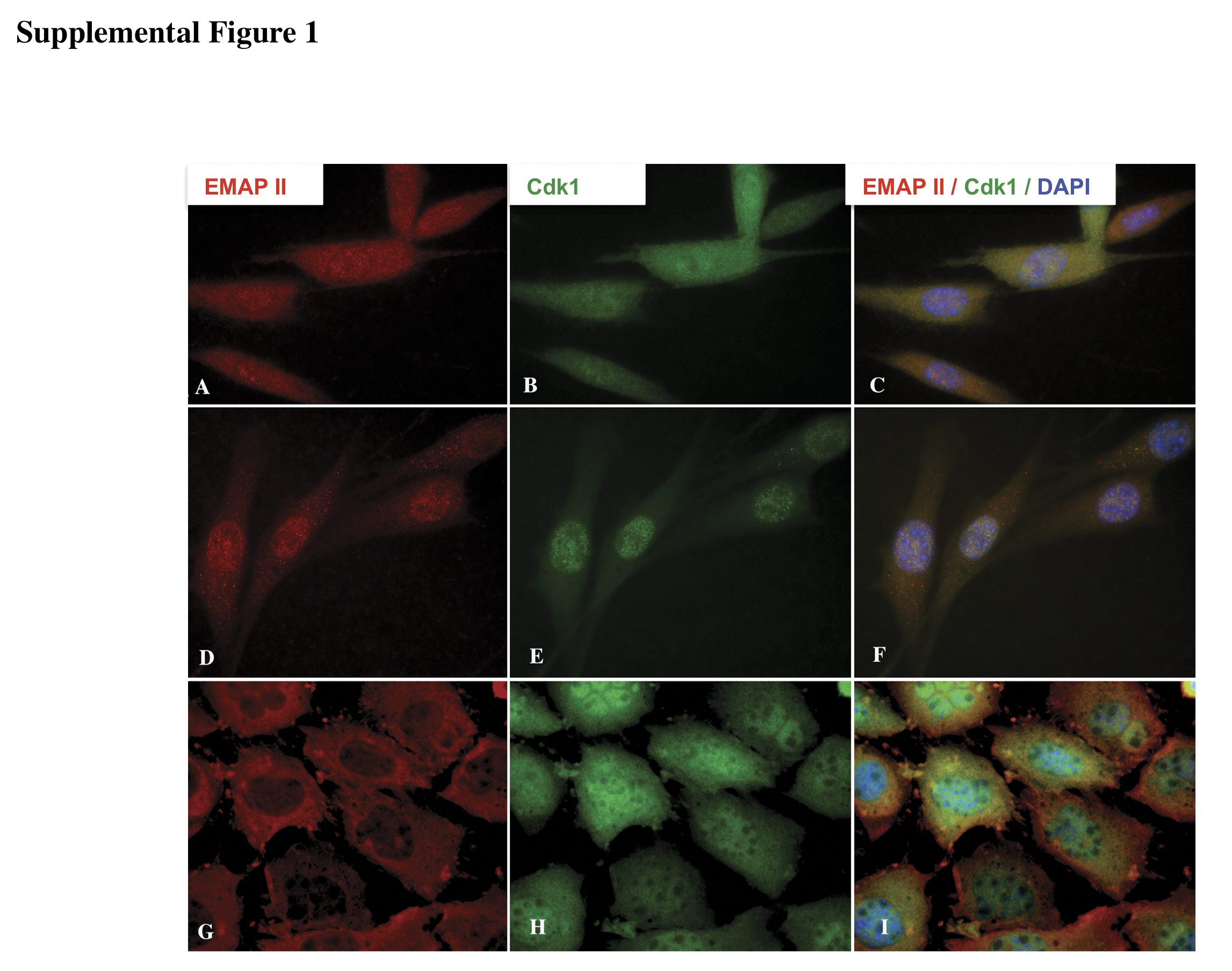

Supplement: Figure S1 — EMAP II and Cdk1 co-localize throughout cell cycle. Distribution of EMAP II and Cdk1 were examined throughout cell cycle using co-immunofluorescence. During G0 (A–C) EMAP II (A) and Cdk1 (B) were expressed in the cytoplasm and nucleus with a transition to a predominate nuclear expression in S phase (D–F). In contrast, during G2M EMAP II is predominately cytoplasmic (G,I) with Cdk1 being both nuclear and cytoplasmic (H,I). Magnification: 600× (these experiments were performed a minimum of 3 times). (TIF) [file pone.0033101.s001.tif]
